# Ventilation-Induced Lung Injury (VILI) in Neonates: Evidence-Based Concepts and Lung-Protective Strategies

**DOI:** 10.3390/jcm11030557

**Published:** 2022-01-22

**Authors:** Renjithkumar Kalikkot Thekkeveedu, Ahmed El-Saie, Varsha Prakash, Lakshmi Katakam, Binoy Shivanna

**Affiliations:** 1 Section of Neonatology, Department of Pediatrics, University of Mississippi Medical Center, Jackson, MS 39216, USA; rkalikkot@umc.edu; 2 Section of Neonatology, Department of Pediatrics, Children’s Mercy Hospital, Kansas City, MO 64106, USA; aelsaie@cmh.edu; 3 Department of Pediatrics, Cairo University, Cairo 11956, Egypt; 4 Department of Pathology, University of Mississippi Medical Center, Jackson, MS 39216, USA; vprakash@umc.edu; 5 Section of Neonatology, Department of Pediatrics, Baylor College of Medicine, Houston, TX 77030, USA; likataka@texaschildrens.org

**Keywords:** volutrauma, atelectrauma, hyperoxia, biotrauma, volume-targeted ventilation, bronchopulmonary dysplasia

## Abstract

Supportive care with mechanical ventilation continues to be an essential strategy for managing severe neonatal respiratory failure; however, it is well known to cause and accentuate neonatal lung injury. The pathogenesis of ventilator-induced lung injury (VILI) is multifactorial and complex, resulting predominantly from interactions between ventilator-related factors and patient-related factors. Importantly, VILI is a significant risk factor for developing bronchopulmonary dysplasia (BPD), the most common chronic respiratory morbidity of preterm infants that lacks specific therapies, causes life-long morbidities, and imposes psychosocial and economic burdens. Studies of older children and adults suggest that understanding how and why VILI occurs is essential to developing strategies for mitigating VILI and its consequences. This article reviews the preclinical and clinical evidence on the pathogenesis and pathophysiology of VILI in neonates. We also highlight the evidence behind various lung-protective strategies to guide clinicians in preventing and attenuating VILI and, by extension, BPD in neonates. Further, we provide a snapshot of future directions that may help minimize neonatal VILI.

## 1. Introduction

Respiratory morbidities and respiratory failure continue to be serious problems in preterm neonates [1,2,3]. The mainstay of management for severe neonatal respiratory failure is supportive care with mechanical ventilation (MV). However, MV by itself may inflict and accentuate lung injury [3,4,5]. This secondary lung damage caused by MV is often called ventilator-induced lung injury (VILI) [6]. VILI is an important risk factor in extremely low birth weight (ELBW) infants for developing bronchopulmonary dysplasia (BPD) [7,8]. Therefore, neonatologists avoid and minimize MV in preterm infants. The use of MV as an initial modality to mitigate respiratory failure in ELBW infants has substantially declined over the past decade [9]. However, MV is at times a life-saving intervention to support the structurally and functionally immature lungs. Some reports indicate that up to ninety-five percent of surviving ELBW infants will be exposed to MV at some point during their NICU stay [10] and 30–80% of them require MV during the first few days of life [11,12], signifying a substantial failure rate of non-invasive respiratory support in the early life of ELBW infants. Thus, there is a need to be aware of lung-protective ventilation strategies.

## 2. Pathology

Acute lung injury caused by MV is characterized by lung inflammation and diffuse alveolar damage [13,14,15]. Increased microvascular permeability from endothelial and epithelial dysfunction and disruption of the alveolar-capillary barrier causes pulmonary edema [16,17]. Histologically, there is diffuse alveolar damage that is characterized by an acute or exudative phase, organizing or proliferative phase, and the late-resolving or fibrotic phase. In patients with repeated episodes of lung injury, all these histological phases may coexist [18,19].

During the acute phase, there is edema, alveolar hemorrhage, hyaline membrane formation, and interstitial widening. This phase is also characterized by necrosis of the endothelial and alveolar cells and extensive thrombus formation [14,20]. Type II pneumocytes hyperplasia occurs towards the end of this phase [15,18,21]. In the organizing phase of VILI, hyaline membranes begin to organize and are incorporated into the alveolar septa through macrophages. There is also granulation tissue development in the alveolar spaces by proliferating myofibroblasts. Type 2 pneumocyte hyperplasia and squamous metaplasia may be more pronounced in this stage. In the late-resolving phase, the granulation tissue incorporates into the alveolar septa, accompanied by fibrosis and hyalinization of the alveolar walls [14,18,19].

Chronic severe VILI in neonates is characterized by inflammation, airway epithelial metaplasia, smooth muscle hypertrophy, and parenchyma fibrosis, which were the typical pathological findings with lung injury in the pre-surfactant era, referred to as old BPD [22]. In the post-surfactant era, neonatal lung injury is mostly mild-to-moderate, characterized by limited lung fibrosis and arrested alveolar and pulmonary vascular development [23].

## 3. Risk Factors and Pathogenesis

MV is an outstanding risk factor for neonatal lung injury. However, it is also important to remember that MV is not the sole contributor of VILI. The pathogenesis of VILI is multifactorial and complex, resulting from interactions between ventilator-related and patient-related factors. Major ventilator-related factors include volutrauma, barotrauma, atelectrauma, oxygen toxicity, and biotrauma. Patient-related factors include immature lungs, surfactant deficiency, asymmetric lung disease, and lung inflammation [24,25,26].

### 3.1. Ventilator-Related Risk Factors

#### 3.1.1. Volutrauma

Volutrauma refers to lung injury caused by exposure to high tidal volume (V_T_) with resultant alveolar overdistension. Lung overinflation will cause overstretching of the small airways and alveoli, resulting in acute edema and increased protein sieving in pulmonary microcirculation [27]. Volutrauma is also associated with the release of proteases, cytokines, and chemokines, leading to the activation of macrophages and neutrophils and increased inflammation and injury in the lungs [28]. Studies have demonstrated that exposure to high V_T_, even for short periods, can induce lung injury [27,29,30]. Volutrauma may also decrease the response to surfactant treatment [29]. The most critical determinant of lung injury appears to be the lung volume at the end of inspiration. A moderately high V_T_ superimposed on a high end-expiratory volume results in end-inspiratory overdistension and volutrauma [31]. Lung injury is more pronounced if the sum of functional residual capacity (FRC) and V_T_ exceeds the total lung capacity (TLC). In preterm neonates, a higher V_T_ delivery is associated with an increased risk of prolonged ventilator dependence [32].

Neonatal clinical studies have demonstrated the importance of preventing volutrauma. Keszler and Abubakar reported that volume targeted ventilation (VTV) stabilizes the delivered V_T_ during MV in preterm infants [33]. Lista et al. demonstrated that VTV could reduce pulmonary inflammation in preterm infants with respiratory distress syndrome (RDS) [34]. Strategies to maintain a constant V_T_ while the lung compliance and resistance changes can decrease volutrauma [35]. The benefits of controlling the V_T_ and limiting volutrauma have been noted in randomized controlled trials (RCTs) that compared VTV to pressure limited ventilation (PLV). A meta-analysis of these studies showed that VTV, compared to PLV, significantly reduced the combined outcome of death or BPD at 36 weeks postmenstrual age (PMA). The incidence of pneumothoraces, hypocarbia, severe intraventricular hemorrhage (IVH), periventricular leukomalacia (PVL), and the mean days of ventilation was also significantly lower in preterm infants supported with VTV than in those supported with PLV [36]. The meta-nalyses and a recent review by Cannavo et al. [37] emphasize that avoiding volutrauma may be important not only for decreasing lung injury, but also brain injury in neonates.

#### 3.1.2. Barotrauma

Barotrauma refers to the lung injury caused by overstretching of the airways and alveoli from exposure to extreme positive pressure. Webb and Tierney were among the first investigators to demonstrate that MV with higher peak pressure produces lung injury in rats [38]. They observed that the severity of the lung injury was directly proportional to the administered peak airway pressure.

The lung expansion is determined by the difference between the alveolar and pleural pressures, i.e., transpulmonary pressure. Currently, there is no reliable technology to accurately measure these pressures. Pleural pressure is difficult to estimate, although esophageal pressures can be used as a surrogate estimate. Plateau pressure is the most commonly used measure for alveolar pressure. Alveolar pressure depends upon the airway resistance and the elastic properties of the lung and chest at any given plateau airway pressure. Therefore, the peak airway pressure may not always be directly proportional to the alveolar or lung distending pressure [6]. This concept is exemplified by the lung physiology of trumpet players. While playing a note, the alveolar pressures of these players can be as high as 150 cm H_2_O; however, at the same time, their pleural pressures can be as high as 140 cm H_2_O and thus, their transpulmonary/lung distending pressure (150 − 140 = 10 cm H_2_O) is low and as a result, their lungs are protected against injury [39]. Other examples include infants with high airway resistance and increased pleural pressures. In both these instances, the transpulmonary pressure and, thereby, the lung injury risk is not increased despite high peak inspiratory pressures. In infants with high airway resistance (e.g., BPD infants) [40,41,42,43], the alveolar pressure is lower than the peak inspiratory pressure because of the increased airway resistance. By contrast, in infants with high pleural pressure (e.g., infants with hydrops, chest wall edema, or abdominal distension), the concept is similar to that of a trumpet player. Although these infants may require a very high inspiratory pressure to accomplish adequate gas exchange, the lung distending or the transpulmonary pressure is not significantly increased because their pleural pressures are also very high.

##### What Is More Injurious: Pressure or Volume?

Whether volutrauma or barotrauma is the more significant determinant of lung injury has been a baffling question. Although volutrauma and barotrauma can be interrelated, studies have demonstrated that more than the high airway pressure per se, it is the degree of lung overinflation that determines the lung injury [44].

In their classic experiment, Dreyfuss et al. [45] subjected rats to high or low V_T_ ventilation using identical high peak airway pressures. Thoraco-abdominal excursions were limited during low-volume ventilation by strapping the chest wall while being exposed to high airway pressure. Increased lung injury was detected in the rats subjected to high V_T_–high airway pressure ventilation, whereas there was no injury in animals ventilated with normal V_T_-high airway pressure ventilation. Similarly, Carlton et al. reported lung injury when preterm lambs were exposed to high V_T_ rather than when exposed to high pressures [27]. Furthermore, systematic analysis of clinical trials in neonates also indicates that VTV causes less lung injury than PLV [36].

#### 3.1.3. Atelectrauma

Atelectrauma results from ventilating at low lung volumes. This type of lung injury results from shear stress secondary to repeated alveolar collapse and expansion (RACE) [8,46,47,48]. The very preterm infants are at high risk for RACE because they have difficulty maintaining their upper airway patency and FRC [49]. Atelectrauma also causes surfactant dysfunction [47].

The role of positive end-expiratory pressure (PEEP) in preventing atelectasis and lung injury cannot be understated. Positive pressure ventilation (PPV) with inadequate PEEP leads to a low residual volume, inability to establish FRC, and atelectasis. Preclinical studies have highlighted the importance of PEEP during ventilation. In rats, Webb and Tierney [38] have demonstrated that MV with very high peak airway pressures and a high PEEP of 10 cm H_2_O resulted in no lung injury, whereas animals ventilated with the same peak airway pressure but zero PEEP had significant injuries. Muscedere et al. [46] studied rat lungs ventilated with physiologic V_T_ at different end-expiratory pressures (above and below inflection pressure or Pinf). The compliance fell dramatically, and lung injury was severe in groups ventilated with a PEEP below Pinf, while there was no change in the group ventilated with PEEP above Pinf. Sandhar et al. investigated the role of PEEP in surfactant-depleted rabbits and reported a significantly lower incidence of hyaline membrane formation in the group ventilated with PEEP, thereby highlighting its role in the prevention of atelectrauma [50].

Ventilation with sufficient end-expiratory lung volume has also been shown to influence exogenous surfactant efficacy [51]. Injury to ventilated surfactant-deficient lungs can be minimized by recruiting alveoli using appropriate mean airway pressures and maintaining lung volume at higher than normal FRC [52]. The lung injury risk during volume recruitment is significantly less than the damage arising from de-recruitment and atelectasis [53]. Mead et al. noted that the stretching forces at the margins between open and collapsed regions of lung parenchyma could be five times more than that observed between the open regions [54].

**Figure 1:**
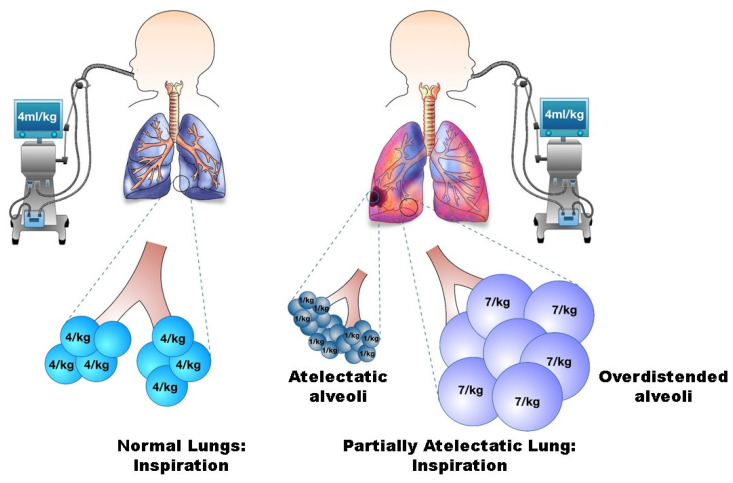
Schema of regional volutrauma while ventilating atelectatic lungs.

Finally, atelectasis can cause regional volutrauma. The delivered V_T_ takes the path of least resistance and preferentially enters and distends the aerated lungs rather than the atelectatic lungs because the critical opening pressure is lower in aerated regions than in the atelectatic regions of the lungs (Laplace’s law). This heterogeneous distribution of V_T_ leads to regional volutrauma and injury of the relatively healthy lungs [5,55] despite the set V_T_ being in the normal range (Figure 1).

#### 3.1.4. Oxidative Stress

Oxygen therapy is frequently used to mitigate hypoxic respiratory failure in infants; however, excessive oxygen exposure (hyperoxia) causes lung injury [56]. Northway et al. initially described BPD as a disorder resulting from lung oxygen toxicity [22]. In newborn guinea pigs exposed to 100% oxygen, they observed radiographic and histologic features similar to BPD infants [57]. Preterm infants are particularly susceptible to oxygen toxicity because the antioxidant mechanisms are not fully developed until the third trimester [5,56,58]. Further, the ability to sequester hyperoxia-mediated generation of reactive oxygen species (ROS) is decreased in neonates [59]. The deleterious effects of hyperoxia are mediated by ROS and lung inflammation. ROS can oxidize cell membrane lipids, proteins, nucleic acid, and enzymes, causing cell death and tissue damage [60].

Human studies emphasize the acute consequences of hyperoxia in neonates. A single-center RCT in infants born between 24- and 28-weeks gestational age (GA) compared higher (90%) versus lower (30%) initial oxygen concentration in the delivery room. Infants who received a higher oxygen load had higher oxidative stress and inflammation and were at higher risk of developing BPD [61]. Similar results were reported in infants between 24- and 34-weeks GA who were randomized at birth to receive either 21% or 100% oxygen and then titrated to achieve a SpO_2_ target between 85% and 94% [62]. Oxidative stress, respiratory morbidities, and BPD rates were lower in the infants initially resuscitated with room air. Trials have also shown that resuscitating asphyxiated newborn infants with room air reduces mortality compared to resuscitation with 100% oxygen [63]. A recent meta-analysis [64] confirmed this finding, leading to neonatal resuscitation guidelines that caution against starting resuscitation with 100% FiO_2_ in term and near-term infants [65,66]. However, the clinical studies have not shown convincing evidence that limiting oxygen exposure can prevent BPD. A meta-analysis comparing high (>50%) and low (<50%) initial oxygen exposures in patients <32 weeks GA found no differences in the BPD rates between the groups [67]. Another larger meta-analysis of infants born at <28 weeks GA examining the effects of low (85–89%) versus high (91–95%) SpO_2_ reported no differences in BPD rates between the groups. However, they observed that the mortality and necrotizing enterocolitis was increased, and severe retinopathy of prematurity was reduced in the low-oxygen saturation-targeted infants [68]. Additionally, the risk of mortality and severe intraventricular hemorrhage is significantly increased in preterm infants if their oxygen saturation remains below 80% in the first 5 min of life [69,70]. These findings suggest the importance of avoiding hypoxia and hyperoxia, both of which can cause oxidative stress, during the neonatal period. The European consensus guidelines recommend targeting oxygen saturation between 90% to 94% in preterm infants receiving oxygen therapy [71].

#### 3.1.5. Biotrauma

Biotrauma results from the release of inflammatory mediators (cytokines and chemokines) secondary to injuries caused by volutrauma, barotrauma, atelectrauma, oxygen toxicity, and sepsis, magnifying the initial injury within the lungs [6]. Inflammatory cells accumulate in the preterm lung during VILI [72], and there is an increased expression of pro-inflammatory cytokines [73,74] and decreased expression of anti-inflammatory cytokines [75]. The inflammatory response from acute lung injury need not be compartmentalized to the lungs [76,77]. The loss of compartmentalization is a two-way disturbance, with cytokines and microbes shifting from the vascular to the alveolar compartment and vice versa [77]. When these mediators are translocated into the systemic circulation, systemic inflammatory response syndrome occurs, causing widespread inflammatory damage and multi-organ dysfunction [78]. Further, biotrauma can cause systemic infection or sepsis when the respiratory tract is infected or colonized with pathogenic microbes [79,80,81,82].

#### 3.1.6. Mechanical Power, Stress and Strain

Mechanical power is the amount of energy transferred from the mechanical ventilator to the lungs per unit of time and is expressed in joules per minute [83]. The risk of VILI is directly proportional to the duration and amount of energy delivered to the lungs [83]. Mechanical power is a function of tidal volume, respiratory rate, and PEEP [83]. Therefore, the amount of power applied to the lungs is dependent on the ventilatory parameters set by the patient care provider team [84]. However, the risk of VILI from mechanical power depends on the lung size and the underlying lung disease. The energy transfer will be less intense if the lungs have a large surface and uniform mechanical properties. However, if the lungs are small or heterogeneous with varying mechanical properties, the risk of VILI is significantly increased for the same mechanical power delivered [85,86,87]. In a recent international multicenter observational study that involved 55 pediatric intensive care units, the use of a higher mechanical power was associated with an increased risk of BPD [88].

Lung stress is defined as force per unit area and is expressed in the same units as pressure [89], whereas lung strain is define as the change in lung volume caused by lung stress [90]. Mechanical power is the major determinant of lung stress and strain [83]. Stress and strain play an important role in mediating VILI in patients with heterogeneous lung disease because stress concentrators arise at margins between atelectatic and aerated lung units [91,92]. Strain and stress are concentrated in these regions because the applied V_T_ preferentially enters, over-distends, and stretches the adjacent normal lungs, which are constrained by the non-expandable atelectatic lungs. This focused stress can be more than two-fold greater than the transpulmonary pressure applied to the whole lung [54,93]. Additionally, the risk for VILI also depends upon whether the lungs are subjected to dynamic or static strain. Lung volume change mediated by V_T_ causes dynamic strain, whereas the volume change mediated by PEEP causes static strain, and dynamic strain is more injurious to the lungs than static strain [94].

### 3.2. Patient-Related Risk Factors

#### 3.2.1. Lung Immaturity

Preterm infants are susceptible to lung injury because their lungs are structurally and functionally immature. Infants with lung immaturity have decreased functional lung units. This means they will need oxygen therapy and MV support to save their lives and prevent brain damage. However, these infants are not equipped with repair mechanisms to counter the adverse effects of these supportive therapies [95,96]. Surfactant-deficient preterm lungs are easily injured during MV [24]. An increased tendency for the collapse of air spaces, the need for higher pressures to recruit and keep the lungs open, and an increase in surface tension, all contribute to lung injury. Preterm lungs are very non-compliant but are supported by a very compliant chest wall. At the same time, the distal airways are highly compliant as they lack smooth muscle and cartilage. This leads to the expansion of distal airways with collapsed alveoli, causing injury to both the airways and atelectatic alveoli [24]. The developmental immaturity of the collagen and elastin components in the respiratory system of preterm infants predisposes them to volutrauma [95,96]. Similarly, prematurity-associated antioxidant enzyme deficiency [5,56,58] increases the risk of ROS-mediated lung injury.

#### 3.2.2. Preexisting Lung Disease

Preexisting lung disease, especially asymmetric lung disease, is a significant risk factor for VILI. When heterogeneous lungs, comprised of both aerated and consolidated regions, are ventilated, the administered V_T_ takes the path of least resistance and preferentially enters and over-distends the aerated or good regions of the lung rather than the consolidated or atelectatic regions because the critical opening pressure is lower in the healthier lungs [97,98]. This heterogeneous V_T_ distribution leads to regional volutrauma of relatively healthy lungs despite the administered V_T_ being in the normal range (Figure 1). Importantly, the existence of asymmetric lung disease may not be easily recognized because chest X-rays, the commonly used diagnostic imaging modality to detect lung disease, are not sensitive enough to diagnose asymmetric lung disease [96].

#### 3.2.3. Nutrition

Good nutrition is necessary for somatic growth and development [99]. Likewise, optimal nutrition is also necessary for lung development and repair [100,101,102,103], as well as defense against infection [42,104,105] and oxidative stress [106,107,108], which are all risk factors for VILI. Preterm infants are at risk of poor nutritional states due to the increased work of breathing, immature gastrointestinal function, fluid restriction, and exposure to medications such as steroids and diuretics [42,109,110]. Intrauterine growth restriction [111] and postnatal growth restriction [112] increase the risk for chronic lung injury. Animal studies suggest that both pre-and post-natal nutritional deficiency are risk factors for lung injury. Nutritional deficiency mediates lung injury by altering the lung surface area–bodyweight ratio, decreasing antioxidant activity [113], delaying type 2 alveolar epithelial cell maturation, reducing surfactant production [113], impairing pulmonary alveolarization and vascular development, causing pulmonary vascular remodeling [114,115], and inducing intestinal dysbiosis [116]. By contrast, human studies suggest that increased caloric intake in the first days of life is associated with decreased respiratory morbidities in preterm infants [117,118].

## 4. Consequences of VILI

**Figure 2:**
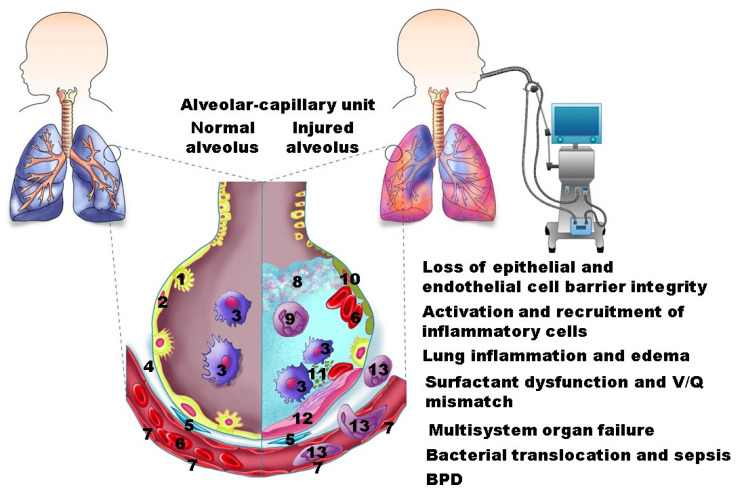
Schema of consequences of VILI: 1: Type II alveolar epithelial cell; 2: Type I alveolar epithelial cell; 3: Alveolar macrophage; 4: Interstitium; 5: Fibroblast; 6: Red blood cell; 7: Endothelial cell; 8: Protein-rich edema; 9: Activated neutrophil; 10: Injured type I alveolar epithelial cell; 11: Cellular debris; 12: Hyaline membrane; 13: Neutrophil.

MV injures lung epithelial and endothelial cells and disrupts alveolarization and vasculogenesis. The damaged blood vessels become leaky, and their endothelial cells become activated, leading to the recruitment of inflammatory cells and the accumulation of inflammatory mediators and protein-rich fluid in the lungs. These inflammatory changes increase the distance between the capillary endothelial cells and alveolar epithelial cells, decreasing the gas-exchange efficacy. These changes also cause surfactant dysfunction and deficiency, hyaline membrane formation, and atelectasis [31,45,96]. The inflammatory mediators and bacterial flora in the lungs can also translocate to the systemic circulation, causing systemic inflammatory response syndrome, sepsis, and multi-organ dysfunction [78]. Finally, severe, recurrent, or persistent lung injury causes suboptimal repair and BPD (Figure 2).

## 5. Lung-Protective Strategies

Studies of adults with acute respiratory distress syndrome (ARDS) suggest that VILI can be mitigated if we employ lung-protective strategies while ventilating respiratory failure patients. The following are some of the strategies to mitigate VILI.

### 5.1. Open Lung Ventilation Strategy

Optimizing lung recruitment and ensuring that the lungs receive even distribution of the delivered V_T_, i.e., open lung concept (OLC), is the fundamental principle of any lung-protective ventilation strategy [119]. Avoiding excessive V_T_ and facilitating uniform distribution of V_T_, and thus decreasing volutrauma and atelectrauma, is the key to preventing acute and chronic lung injury.

### 5.2. Preventing Volutrauma

As discussed in the VILI pathogenesis, decreasing alveolar overdistension by targeting V_T_ attenuates VILI. V_T_ is better regulated and stabilized by VTV than PLV. In PLV, V_T_ delivery can vary significantly depending on the compliance and resistance of the respiratory system and the patient’s effort, resulting in volutrauma or atelectrauma. Studies in both adult and newborn animal models have shown that reducing V_T_ and applying PEEP during conventional MV attenuates VILI. Although PEEP can reduce the severity of VILI, PEEP might favor hyperinflation if V_T_ is not optimized because the main determinant of acute lung injury is end-inspiratory and end-expiratory lung volume [31,46,120]. MV with lower V_T_ has been shown to decrease mortality and the number of days of ventilation compared to higher V_T_ strategies in ARDS [121]. Similarly, a Cochrane review of twenty RCTs comprising more than 1000 infants reported that the VTV reduced the primary outcome of death or BPD at 36 weeks PMA and the secondary outcomes of pneumothorax, MV duration, and the incidence of hypocarbia, severe IVH, and PVL [36].

### 5.3. High-Frequency Ventilation

Animal models of acute lung injury have demonstrated that high-frequency ventilation (HFV) may decrease lung inflammation and improve lung function, mechanics, and histopathology [51,52]. Multiple RCTs compared elective HFV with conventional MV in preterm infants with respiratory failure. Although the systematic reviews have reported that BPD risk in survivors was significantly reduced with HFV use, this effect was inconsistent across studies [122,123,124]. Lung-protective strategies with conventional ventilation may be as good as HFV in preterm infants. Conventional ventilation with optimization of lung volumes can achieve similar degrees of lung protection as HFV [125,126,127].

### 5.4. Preventing and Reversing Atelectrauma

Avoiding atelectrauma by using sufficient inflation pressure to recruit collapsed alveoli and stabilizing the recruited alveoli by sufficient PEEP protects against lung injury. CPAP or PEEP can improve respiratory function by: (a) reducing upper airway resistance by mechanically splinting the airway [128]; (b) increasing FRC [128]; (c) reducing inspiratory resistance by dilating the airways [129]; (d) increasing lung compliance; (e) stabilizing the chest wall; (f) increasing the mean airway pressure and improving the ventilation-perfusion mismatch [130]; and (g) conserving surfactant [131].

### 5.5. Noninvasive Respiratory Support

Three landmark studies compared CPAP to MV. In the COIN trial, Morley et al. randomized 610 infants between 25 and 28 6/7 weeks GA to initial respiratory management with either CPAP or MV. CPAP decreased the days on MV and the requirement for oxygen at 28 days. However, they did not find any significant difference in the rates of death or BPD between the groups [132]. The SUPPORT trial enrolled 1316 infants between 24 and 27 6/7 weeks GA and randomized them either to initial CPAP therapy with subsequent selective rescue surfactant therapy or to primary MV with prophylactic surfactant therapy. There was no significant difference in the rates of death or BPD between the groups. However, infants randomized to CPAP treatment required less frequent intubation, fewer days of MV, and decreased postnatal corticosteroid therapy [133]. In the Vermont Oxford Network trial, Dunn et al. randomized 648 infants between 26 and 29 6/7 weeks GA to prophylactic surfactant followed by MV, prophylactic surfactant followed by extubation to CPAP, or initial CPAP therapy with selective surfactant treatment [134]. The rates of death or BPD at 36 weeks PMA between the groups were not different, although early CPAP reduced the need for intubation and surfactant treatment.

A meta-analysis comparing early surfactant administration with brief MV followed by extubation to CPAP vs. selective surfactant administration followed by MV and extubation from low respiratory support showed that the former treatment strategy was associated with a reduced need for MV, lower BPD incidence, and lower air leak syndromes [135]. Similarly, a more recent meta-analysis of four RCTs comparing nasal CPAP versus MV in preterm infants reported that CPAP reduces the combined outcome of death or BPD, or both, at 36 weeks PMA [136]. Based on these studies, the Committee on Fetus and Newborn by the American Academy of Pediatrics published a policy statement according to which the early use of CPAP with subsequent selective surfactant administration in preterm infants results in lower rates of BPD/death compared to prophylactic or early surfactant therapy [137].

Other forms of nasal ventilatory strategies (nasal intermittent MV (NIMV) or nasal intermittent positive pressure ventilation (NIPPV)) have also been shown to protect the lungs. Kugelman et al. randomized 84 preterm infants to either nasal CPAP or NIMV and observed that infants randomized to NIMV were intubated less often and had decreased BPD incidence [138]. Another retrospective study of preterm infants weighing ≤1250 g assessed the impact of CPAP versus synchronized NIPPV (SNIPPV). In the cohort of infants with birth weights between 500 and 750 g, NIPPV was associated with reduced incidence of the combined outcome of BPD and death compared to CPAP [139]. In a prospective randomized study, Bhandari et al. compared SNIPPV with conventional MV in 41 infants with a birth weight between 600 and 1250 g. They found a lower BPD and death rate in the SNIPPV group [140]. A meta-analysis of three studies including 360 infants concluded that NIPPV was superior to CPAP in preventing invasive ventilation, although neither of these therapies decreased BPD [141]. These results were substantiated by a larger multinational RCT in ELBW infants, which showed no difference in BPD-free survival at 36 weeks PMA with either CPAP or NIPPV [142]. However, a recent meta-analysis indicates that when compared to CPAP, ventilator-generated synchronized NIPPV may decrease BPD incidence in preterm infants [143].

## 6. Conclusions

**Figure 3:**
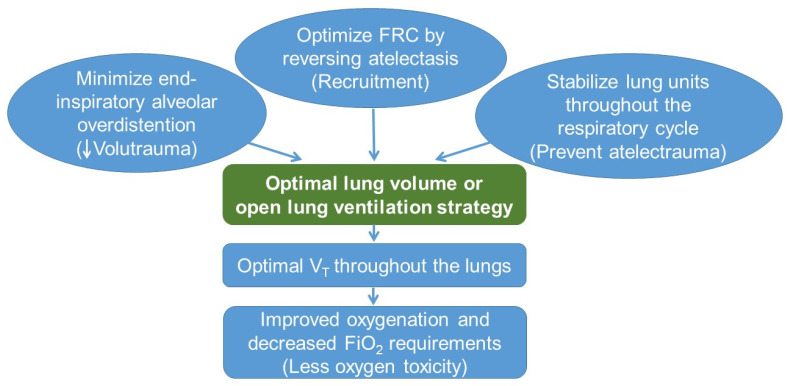
Summary of lung-protective strategies: FRC: Functional residual capacity; V_T_: Tidal volume; FiO_2_: Fraction of inspired oxygen.

MV is a key risk factor for both acute and chronic lung injury in neonates. Although the pathogenesis of VILI is complex and multifactorial, it is possible to prevent or mitigate this injury in many neonates. Importantly, the emphasis should be to avoid MV and use noninvasive respiratory support if possible as the initial modality to manage respiratory failure in neonates, especially ELBW infants. The fundamental principle is to achieve an acceptable but not entirely normal level of gas exchange with the least deleterious form of ventilatory support. If MV is a necessity, employment of lung-protective ventilatory strategies should be considered. End-inspiratory alveolar overdistension should be minimized by employing VTV. FRC should be optimized by reversing atelectasis and stabilizing lung units by providing sufficient PEEP or CPAP. Incorporating these three strategies will enable the delivery of optimal V_T_ uniformly throughout the lungs, improving oxygenation and simultaneously minimizing volutrauma, atelectrauma, and oxygen toxicity (Figure 3).

## 7. Future Directions

Although not comprehensive, this article discusses two important concepts that can help prevent or mitigate VILI in neonates. Estimating the mechanical power needed to support the lung function can be an important strategy to decrease mechanical energy delivered to the lungs over time by MV and the resultant lung injury. The mechanical power needed during MV can be estimated by measuring the patient’s respiratory mechanics, such as pulmonary and respiratory system elastance. Second, normalizing the delivered V_T_ to lung size rather than bodyweight is necessary to provide accurate respiratory support and prevent or decrease VILI. The aerated lung size or FRC can be measured by quantitative CT scan analysis or gas dilution techniques.

## Data Availability

Not applicable.

## References

[1] Chami M., Geoffray A. (1997). Pulmonary sequelae of prematurity: Radiological manifestations. Pediatr. Pulmonol. Suppl..

[2] Flor-de-Lima F., Rocha G., Guimarães H. (2012). Impact of changes in perinatal care on neonatal respiratory outcome and survival of preterm newborns: An overview of 15 years. Crit. Care Res. Pract..

[3] Sandri F., Plavka R., Ancora G., Simeoni U., Stranak Z., Martinelli S., Mosca F., Nona J., Thomson M., Verder H. (2010). Prophylactic or early selective surfactant combined with nCPAP in very preterm infants. Pediatrics.

[4] Artigas A., Bernard G.R., Carlet J., Dreyfuss D., Gattinoni L., Hudson L., Lamy M., Marini J.J., Matthay M.A., Pinsky M.R. (1998). The American-European Consensus Conference on ARDS, part 2. Ventilatory, pharmacologic, supportive therapy, study design strategies and issues related to recovery and remodeling. Intensive Care Med..

[5] Clark R.H., Gerstmann D.R., Jobe A.H., Moffitt S.T., Slutsky A.S., Yoder B.A. (2001). Lung injury in neonates: Causes, strategies for prevention, and long-term consequences. J. Pediatr..

[6] Slutsky A.S., Ranieri V.M. (2013). Ventilator-induced lung injury. N. Engl. J. Med..

[7] Kalikkot Thekkeveedu R., Guaman M.C., Shivanna B. (2017). Bronchopulmonary dysplasia: A review of pathogenesis and pathophysiology. Respir. Med..

[8] Jobe A.H., Ikegami M. (1998). Mechanisms initiating lung injury in the preterm. Early Hum. Dev..

[9] Hatch L.D., Clark R.H., Carlo W.A., Stark A.R., Ely E.W., Patrick S.W. (2021). Changes in Use of Respiratory Support for Preterm Infants in the US, 2008–2018. JAMA Pediatr..

[10] Keszler M., Sant’Anna G. (2015). Mechanical Ventilation and Bronchopulmonary Dysplasia. Clin. Perinatol..

[11] Stoll B.J., Hansen N.I., Bell E.F., Walsh M.C., Carlo W.A., Shankaran S., Laptook A.R., Sánchez P.J., Van Meurs K.P., Wyckoff M. (2015). Trends in Care Practices, Morbidity, and Mortality of Extremely Preterm Neonates, 1993–2012. JAMA.

[12] Shi Y., Muniraman H., Biniwale M., Ramanathan R. (2020). A Review on Non-invasive Respiratory Support for Management of Respiratory Distress in Extremely Preterm Infants. Front. Pediatr..

[13] Joelsson J.P., Ingthorsson S., Kricker J., Gudjonsson T., Karason S. (2021). Ventilator-induced lung-injury in mouse models: Is there a trap?. Lab. Anim. Res..

[14] Matute-Bello G., Downey G., Moore B.B., Groshong S.D., Matthay M.A., Slutsky A.S., Kuebler W.M. (2011). An official American Thoracic Society workshop report: Features and measurements of experimental acute lung injury in animals. Am. J. Respir. Cell Mol. Biol..

[15] Thille A.W., Esteban A., Fernández-Segoviano P., Rodriguez J.M., Aramburu J.A., Peñuelas O., Cortés-Puch I., Cardinal-Fernández P., Lorente J.A., Frutos-Vivar F. (2013). Comparison of the Berlin definition for acute respiratory distress syndrome with autopsy. Am. J. Respir. Crit. Care Med..

[16] Jin S., Ding X., Yang C., Li W., Deng M., Liao H., Lv X., Pitt B.R., Billiar T.R., Zhang L.M. (2021). Mechanical Ventilation Exacerbates Poly (I:C) Induced Acute Lung Injury: Central Role for Caspase-11 and Gut-Lung Axis. Front. Immunol..

[17] Chun C.D., Liles W.C., Frevert C.W., Glenny R.W., Altemeier W.A. (2010). Mechanical ventilation modulates Toll-like receptor-3-induced lung inflammation via a MyD88-dependent, TLR4-independent pathway: A controlled animal study. BMC Pulm. Med..

[18] Tomashefski J.F. (2000). Pulmonary pathology of acute respiratory distress syndrome. Clin. Chest Med..

[19] Hughes K.T., Beasley M.B. (2017). Pulmonary Manifestations of Acute Lung Injury: More Than Just Diffuse Alveolar Damage. Arch. Pathol. Lab. Med..

[20] Bachofen M., Weibel E.R. (1982). Structural alterations of lung parenchyma in the adult respiratory distress syndrome. Clin. Chest Med..

[21] Deptula N., Royse E., Kemp M.W., Miura Y., Kallapur S.G., Jobe A.H., Hillman N.H. (2016). Brief mechanical ventilation causes differential epithelial repair along the airways of fetal, preterm lambs. Am. J. Physiol. Lung Cell. Mol. Physiol..

[22] Northway W.H., Rosan R.C., Porter D.Y. (1967). Pulmonary disease following respirator therapy of hyaline-membrane disease. Bronchopulmonary dysplasia. N. Engl. J. Med..

[23] Husain A.N., Siddiqui N.H., Stocker J.T. (1998). Pathology of arrested acinar development in postsurfactant bronchopulmonary dysplasia. Hum. Pathol..

[24] Coker P.J., Hernandez L.A., Peevy K.J., Adkins K., Parker J.C. (1992). Increased sensitivity to mechanical ventilation after surfactant inactivation in young rabbit lungs. Crit. Care Med..

[25] Ikegami M., Kallapur S.G., Jobe A.H. (2004). Initial responses to ventilation of premature lambs exposed to intra-amniotic endotoxin 4 days before delivery. Am. J. Physiol. Lung Cell Mol. Physiol..

[26] Altemeier W.A., Matute-Bello G., Frevert C.W., Kawata Y., Kajikawa O., Martin T.R., Glenny R.W. (2004). Mechanical ventilation with moderate tidal volumes synergistically increases lung cytokine response to systemic endotoxin. Am. J. Physiol. Lung Cell Mol. Physiol..

[27] Carlton D.P., Cummings J.J., Scheerer R.G., Poulain F.R., Bland R.D. (1990). Lung overexpansion increases pulmonary microvascular protein permeability in young lambs. J. Appl. Physiol. 1985.

[28] Mokres L.M., Parai K., Hilgendorff A., Ertsey R., Alvira C.M., Rabinovitch M., Bland R.D. (2010). Prolonged mechanical ventilation with air induces apoptosis and causes failure of alveolar septation and angiogenesis in lungs of newborn mice. Am. J. Physiol. Lung Cell Mol. Physiol..

[29] Wada K., Jobe A.H., Ikegami M. (1997). Tidal volume effects on surfactant treatment responses with the initiation of ventilation in preterm lambs. J. Appl. Physiol. 1985.

[30] Bjorklund L.J., Ingimarsson J., Curstedt T., John J., Robertson B., Werner O., Vilstrup C.T. (1997). Manual ventilation with a few large breaths at birth compromises the therapeutic effect of subsequent surfactant replacement in immature lambs. Pediatr. Res..

[31] Dreyfuss D., Saumon G. (1998). Ventilator-induced lung injury: Lessons from experimental studies. Am. J. Respir. Crit. Care Med..

[32] Ali K., Kagalwalla S., Cockar I., Williams E.E., Tamura K., Dassios T., Greenough A. (2019). Prediction of prolonged ventilator dependence in preterm infants. Eur. J. Pediatr..

[33] Keszler M., Abubakar K. (2004). Volume guarantee: Stability of tidal volume and incidence of hypocarbia. Pediatr. Pulmonol..

[34] Lista G., Colnaghi M., Castoldi F., Condo V., Reali R., Compagnoni G., Mosca F. (2004). Impact of targeted-volume ventilation on lung inflammatory response in preterm infants with respiratory distress syndrome (RDS). Pediatr. Pulmonol..

[35] Jain D., Bancalari E. (2019). New Developments in Respiratory Support for Preterm Infants. Am. J. Perinatol..

[36] Klingenberg C., Wheeler K.I., McCallion N., Morley C.J., Davis P.G. (2017). Volume-targeted versus pressure-limited ventilation in neonates. Cochrane Database Syst. Rev..

[37] Cannavò L., Rulli I., Falsaperla R., Corsello G., Gitto E. (2020). Ventilation, oxidative stress and risk of brain injury in preterm newborn. Ital. J. Pediatr..

[38] Webb H.H., Tierney D.F. (1974). Experimental pulmonary edema due to intermittent positive pressure ventilation with high inflation pressures. Protection by positive end-expiratory pressure. Am. Rev. Respir. Dis..

[39] Bouhuys A. (1969). Physiology and musical instruments. Nature.

[40] Vanhaverbeke K., Slaats M., Al-Nejar M., Everaars N., Snoeckx A., Spinhoven M., El Addouli H., Lauwers E., Van Eyck A., De Winter B.Y. (2021). Functional respiratory imaging provides novel insights into the long-term respiratory sequelae of bronchopulmonary dysplasia. Eur. Respir. J..

[41] Schmidt A.R., Ramamoorthy C. (2021). Bronchopulmonary dysplasia. Paediatr. Anaesth..

[42] McEvoy C.T., Jain L., Schmidt B., Abman S., Bancalari E., Aschner J.L. (2014). Bronchopulmonary dysplasia: NHLBI Workshop on the Primary Prevention of Chronic Lung Diseases. Ann. Am. Thorac. Soc..

[43] Manti S., Galdo F., Parisi G.F., Napolitano M., Decimo F., Leonardi S., Miraglia Del Giudice M. (2021). Long-term effects of bronchopulmonary dysplasia on lung function: A pilot study in preschool children’s cohort. J. Asthma Off. J. Assoc. Care Asthma.

[44] Rahn H., Otis A.B., Chadwick L.E., Fenn W.O. (1946). The pressure-volume diagram of the thorax and lung. Am. J. Physiol..

[45] Dreyfuss D., Soler P., Basset G., Saumon G. (1988). High inflation pressure pulmonary edema. Respective effects of high airway pressure, high tidal volume, and positive end-expiratory pressure. Am. Rev. Respir. Dis..

[46] Muscedere J.G., Mullen J.B., Gan K., Slutsky A.S. (1994). Tidal ventilation at low airway pressures can augment lung injury. Am. J. Respir. Crit. Care Med..

[47] Taskar V., John J., Evander E., Robertson B., Jonson B. (1997). Surfactant dysfunction makes lungs vulnerable to repetitive collapse and reexpansion. Am. J. Respir. Crit. Care Med..

[48] Williams E.E., Greenough A. (2021). Lung Protection During Mechanical Ventilation in the Premature Infant. Clin. Perinatol..

[49] Morley C. (1999). Continuous distending pressure. Arch. Dis. Child. Fetal Neonatal Ed..

[50] Sandhar B.K., Niblett D.J., Argiras E.P., Dunnill M.S., Sykes M.K. (1988). Effects of positive end-expiratory pressure on hyaline membrane formation in a rabbit model of the neonatal respiratory distress syndrome. Intensive Care Med..

[51] Froese A.B., McCulloch P.R., Sugiura M., Vaclavik S., Possmayer F., Moller F. (1993). Optimizing alveolar expansion prolongs the effectiveness of exogenous surfactant therapy in the adult rabbit. Am. Rev. Respir. Dis..

[52] McCulloch P.R., Forkert P.G., Froese A.B. (1988). Lung volume maintenance prevents lung injury during high frequency oscillatory ventilation in surfactant-deficient rabbits. Am. Rev. Respir. Dis..

[53] Bond D.M., Froese A.B. (1993). Volume recruitment maneuvers are less deleterious than persistent low lung volumes in the atelectasis-prone rabbit lung during high-frequency oscillation. Crit. Care Med..

[54] Mead J., Takishima T., Leith D. (1970). Stress distribution in lungs: A model of pulmonary elasticity. J. Appl. Physiol..

[55] Berger T.M., Fontana M., Stocker M. (2013). The journey towards lung protective respiratory support in preterm neonates. Neonatology.

[56] Mathias M., Chang J., Perez M., Saugstad O. (2021). Supplemental Oxygen in the Newborn: Historical Perspective and Current Trends. Antioxidants.

[57] Northway W.H., Rosan R.C., Shahinian L., Castellino R.A., Gyepes M.T., Durbridge T. (1969). Radiologic and histologic investigation of pulmonary oxygen toxicity in newborn guinea pigs. Invest. Radiol..

[58] Frank L. (1992). Antioxidants, nutrition, and bronchopulmonary dysplasia. Clin. Perinatol..

[59] Berkelhamer S.K., Kim G.A., Radder J.E., Wedgwood S., Czech L., Steinhorn R.H., Schumacker P.T. (2013). Developmental differences in hyperoxia-induced oxidative stress and cellular responses in the murine lung. Free Radic. Biol. Med..

[60] Cannavò L., Perrone S., Viola V., Marseglia L., Di Rosa G., Gitto E. (2021). Oxidative Stress and Respiratory Diseases in Preterm Newborns. Int. J. Mol. Sci..

[61] Vento M., Moro M., Escrig R., Arruza L., Villar G., Izquierdo I., Roberts L.J., Arduini A., Escobar J.J., Sastre J. (2009). Preterm resuscitation with low oxygen causes less oxidative stress, inflammation, and chronic lung disease. Pediatrics.

[62] Kapadia V.S., Chalak L.F., Sparks J.E., Allen J.R., Savani R.C., Wyckoff M.H. (2013). Resuscitation of preterm neonates with limited versus high oxygen strategy. Pediatrics.

[63] Davis P.G., Tan A., O’Donnell C.P., Schulze A. (2004). Resuscitation of newborn infants with 100% oxygen or air: A systematic review and meta-analysis. Lancet.

[64] Welsford M., Nishiyama C., Shortt C., Isayama T., Dawson J.A., Weiner G., Roehr C.C., Wyckoff M.H., Rabi Y. (2019). Room Air for Initiating Term Newborn Resuscitation: A Systematic Review With Meta-analysis. Pediatrics.

[65] Escobedo M.B., Aziz K., Kapadia V.S., Lee H.C., Niermeyer S., Schmölzer G.M., Szyld E., Weiner G.M., Wyckoff M.H., Yamada N.K. (2020). 2019 American Heart Association Focused Update on Neonatal Resuscitation: An Update to the American Heart Association Guidelines for Cardiopulmonary Resuscitation and Emergency Cardiovascular Care. Pediatrics.

[66] Wyckoff M.H., Wyllie J., Aziz K., de Almeida M.F., Fabres J., Fawke J., Guinsburg R., Hosono S., Isayama T., Kapadia V.S. (2020). Neonatal Life Support: 2020 International Consensus on Cardiopulmonary Resuscitation and Emergency Cardiovascular Care Science With Treatment Recommendations. Circulation.

[67] Brown J.V., Moe-Byrne T., Harden M., McGuire W. (2012). Lower versus higher oxygen concentration for delivery room stabilisation of preterm neonates: Systematic review. PLoS ONE.

[68] Saugstad O.D., Aune D. (2014). Optimal oxygenation of extremely low birth weight infants: A meta-analysis and systematic review of the oxygen saturation target studies. Neonatology.

[69] Thamrin V., Saugstad O.D., Tarnow-Mordi W., Wang Y.A., Lui K., Wright I.M., De Waal K., Travadi J., Smyth J.P., Craven P. (2018). Preterm Infant Outcomes after Randomization to Initial Resuscitation with FiO_2_ 0.21 or 1.0. J. Pediatr..

[70] Kapadia V., Oei J.L., Finer N., Rich W., Rabi Y., Wright I.M., Rook D., Vermeulen M.J., Tarnow-Mordi W.O., Smyth J.P. (2021). Outcomes of delivery room resuscitation of bradycardic preterm infants: A retrospective cohort study of randomised trials of high vs low initial oxygen concentration and an individual patient data analysis. Resuscitation.

[71] Sweet D.G., Carnielli V., Greisen G., Hallman M., Ozek E., Te Pas A., Plavka R., Roehr C.C., Saugstad O.D., Simeoni U. (2019). European Consensus Guidelines on the Management of Respiratory Distress Syndrome—2019 Update. Neonatology.

[72] Vogel E.R., Britt R.D., Trinidad M.C., Faksh A., Martin R.J., MacFarlane P.M., Pabelick C.M., Prakash Y.S. (2015). Perinatal oxygen in the developing lung. Can. J. Physiol. Pharmacol..

[73] Naik A.S., Kallapur S.G., Bachurski C.J., Jobe A.H., Michna J., Kramer B.W., Ikegami M. (2001). Effects of ventilation with different positive end-expiratory pressures on cytokine expression in the preterm lamb lung. Am. J. Respir. Crit. Care Med..

[74] Tremblay L.N., Miatto D., Hamid Q., Govindarajan A., Slutsky A.S. (2002). Injurious ventilation induces widespread pulmonary epithelial expression of tumor necrosis factor-alpha and interleukin-6 messenger RNA. Crit. Care Med..

[75] Bohrer B., Silveira R.C., Neto E.C., Procianoy R.S. (2010). Mechanical ventilation of newborns infant changes in plasma pro- and anti-inflammatory cytokines. J. Pediatr..

[76] Chiumello D., Pristine G., Slutsky A.S. (1999). Mechanical ventilation affects local and systemic cytokines in an animal model of acute respiratory distress syndrome. Am. J. Respir. Crit. Care Med..

[77] Haitsma J.J., Uhlig S., Goggel R., Verbrugge S.J., Lachmann U., Lachmann B. (2000). Ventilator-induced lung injury leads to loss of alveolar and systemic compartmentalization of tumor necrosis factor-alpha. Intensive Care Med..

[78] Curley G.F., Laffey J.G., Zhang H., Slutsky A.S. (2016). Biotrauma and Ventilator-Induced Lung Injury: Clinical Implications. Chest.

[79] Nahum A., Hoyt J., Schmitz L., Moody J., Shapiro R., Marini J.J. (1997). Effect of mechanical ventilation strategy on dissemination of intratracheally instilled *Escherichia coli* in dogs. Crit. Care Med..

[80] Verbrugge S.J., Sorm V., van ’t Veen A., Mouton J.W., Gommers D., Lachmann B. (1998). Lung overinflation without positive end-expiratory pressure promotes bacteremia after experimental *Klebsiella pneumoniae* inoculation. Intensive Care Med..

[81] Cakar N., Akinci O., Tugrul S., Ozcan P.E., Esen F., Eraksoy H., Cagatay A., Telci L., Nahum A. (2002). Recruitment maneuver: Does it promote bacterial translocation?. Crit. Care Med..

[82] Ozcan P.E., Cakar N., Tugrul S., Akinci O., Cagatay A., Yilmazbayhan D., Esen F., Telci L., Akpir K. (2007). The effects of airway pressure and inspiratory time on bacterial translocation. Anesth. Analg..

[83] Vasques F., Duscio E., Cipulli F., Romitti F., Quintel M., Gattinoni L. (2018). Determinants and Prevention of Ventilator-Induced Lung Injury. Crit. Care Clin..

[84] Cruz F.F., Ball L., Rocco P.R.M., Pelosi P. (2018). Ventilator-induced lung injury during controlled ventilation in patients with acute respiratory distress syndrome: Less is probably better. Expert Rev. Respir. Med..

[85] Cressoni M., Chiumello D., Chiurazzi C., Brioni M., Algieri I., Gotti M., Nikolla K., Massari D., Cammaroto A., Colombo A. (2016). Lung inhomogeneities, inflation and [18F]2-fluoro-2-deoxy-D-glucose uptake rate in acute respiratory distress syndrome. Eur. Respir. J..

[86] Cressoni M., Gotti M., Chiurazzi C., Massari D., Algieri I., Amini M., Cammaroto A., Brioni M., Montaruli C., Nikolla K. (2016). Mechanical Power and Development of Ventilator-induced Lung Injury. Anesthesiology.

[87] Silva P.L., Ball L., Rocco P.R.M., Pelosi P. (2019). Power to mechanical power to minimize ventilator-induced lung injury?. Intensive Care Med. Exp..

[88] Bhalla A.K., Klein M.J., Modesto I.A.V., Emeriaud G., Kneyber M.C.J., Medina A., Cruces P., Diaz F., Takeuchi M., Maddux A.B. (2022). Mechanical power in pediatric acute respiratory distress syndrome: A PARDIE study. Crit. Care.

[89] Hubmayr R.D., Kallet R.H. (2018). Understanding Pulmonary Stress-Strain Relationships in Severe ARDS and Its Implications for Designing a Safer Approach to Setting the Ventilator. Respir. Care.

[90] Nieman G.F., Satalin J., Andrews P., Habashi N.M., Gatto L.A. (2016). Lung stress, strain, and energy load: Engineering concepts to understand the mechanism of ventilator-induced lung injury (VILI). Intensive Care Med. Exp..

[91] Makiyama A.M., Gibson L.J., Harris R.S., Venegas J.G. (2014). Stress concentration around an atelectatic region: A finite element model. Respir. Physiol. Neurobiol..

[92] Marini J.J. (2019). Evolving concepts for safer ventilation. Crit. Care.

[93] Cressoni M., Cadringher P., Chiurazzi C., Amini M., Gallazzi E., Marino A., Brioni M., Carlesso E., Chiumello D., Quintel M. (2014). Lung inhomogeneity in patients with acute respiratory distress syndrome. Am. J. Respir. Crit. Care Med..

[94] Protti A., Andreis D.T., Milesi M., Iapichino G.E., Monti M., Comini B., Pugni P., Melis V., Santini A., Dondossola D. (2015). Lung anatomy, energy load, and ventilator-induced lung injury. Intensive Care Med. Exp..

[95] Jobe A.H., Ikegami M. (2000). Lung development and function in preterm infants in the surfactant treatment era. Annu. Rev. Physiol..

[96] Whitehead T., Slutsky A.S. (2002). The pulmonary physician in critical care * 7: Ventilator induced lung injury. Thorax.

[97] Rouby J.J., Lherm T., Martin de Lassale E., Poète P., Bodin L., Finet J.F., Callard P., Viars P. (1993). Histologic aspects of pulmonary barotrauma in critically ill patients with acute respiratory failure. Intensive Care Med..

[98] Goldstein I., Bughalo M.T., Marquette C.H., Lenaour G., Lu Q., Rouby J.J. (2001). Mechanical ventilation-induced air-space enlargement during experimental pneumonia in piglets. Am. J. Respir. Crit. Care Med..

[99] Haschke F., Binder C., Huber-Dangl M., Haiden N. (2019). Early-Life Nutrition, Growth Trajectories, and Long-Term Outcome. Nestle Nutr. Inst. Workshop Ser..

[100] Uberos J., Lardón-Fernández M., Machado-Casas I., Molina-Oya M., Narbona-López E. (2016). Nutrition in extremely low birth weight infants: Impact on bronchopulmonary dysplasia. Minerva Pediatrica.

[101] Klevebro S., Westin V., Stoltz Sjöström E., Norman M., Domellöf M., Edstedt Bonamy A.K., Hallberg B. (2019). Early energy and protein intakes and associations with growth, BPD, and ROP in extremely preterm infants. Clin. Nutr..

[102] Kuiper-Makris C., Selle J., Nüsken E., Dötsch J., Alejandre Alcazar M.A. (2021). Perinatal Nutritional and Metabolic Pathways: Early Origins of Chronic Lung Diseases. Front. Med..

[103] Bancalari E., Jain D. (2019). Bronchopulmonary Dysplasia: 50 Years after the Original Description. Neonatology.

[104] Nadimpalli M.L., Bourke C.D., Robertson R.C., Delarocque-Astagneau E., Manges A.R., Pickering A.J. (2020). Can breastfeeding protect against antimicrobial resistance?. BMC Med..

[105] Bæk O., Ren S., Brunse A., Sangild P.T., Nguyen D.N. (2020). Impaired Neonatal Immunity and Infection Resistance Following Fetal Growth Restriction in Preterm Pigs. Front. Immunol..

[106] Chen J.H., Cottrell E.C., Ozanne S.E. (2010). Early growth and ageing. Nestle Nutr. Workshop Ser. Paediatr. Programme.

[107] Chen Y., Fantuzzi G., Schoeny M., Meier P., Patel A.L. (2019). High-Dose Human Milk Feedings Decrease Oxidative Stress in Premature Infant. JPEN J. Parenter. Enter. Nutr..

[108] Cai C., Zhang Z., Morales M., Wang Y., Khafipour E., Friel J. (2019). Feeding practice influences gut microbiome composition in very low birth weight preterm infants and the association with oxidative stress: A prospective cohort study. Free Radic. Biol. Med..

[109] Poindexter B.B., Martin C.R. (2015). Impact of Nutrition on Bronchopulmonary Dysplasia. Clin. Perinatol..

[110] Aschner J.L., Bancalari E.H., McEvoy C.T. (2017). Can We Prevent Bronchopulmonary Dysplasia?. J. Pediatr..

[111] Pierro M., Villamor-Martinez E., van Westering-Kroon E., Alvarez-Fuente M., Abman S.H., Villamor E. (2021). Association of the dysfunctional placentation endotype of prematurity with bronchopulmonary dysplasia: A systematic review, meta-analysis and meta-regression. Thorax.

[112] Underwood M.A., Lakshminrusimha S., Steinhorn R.H., Wedgwood S. (2021). Malnutrition, poor post-natal growth, intestinal dysbiosis and the developing lung. J. Perinatol. Off. J. Calif. Perinat. Assoc..

[113] Frank L., Lewis P.L., Garcia-Pons T. (1985). Intrauterine growth-retarded rat pups show increased susceptibility to pulmonary O_2_ toxicity. Pediatr. Res..

[114] Rozance P.J., Seedorf G.J., Brown A., Roe G., O’Meara M.C., Gien J., Tang J.R., Abman S.H. (2011). Intrauterine growth restriction decreases pulmonary alveolar and vessel growth and causes pulmonary artery endothelial cell dysfunction in vitro in fetal sheep. Am. J. Physiol. Lung Cell Mol. Physiol..

[115] Wedgwood S., Warford C., Agvateesiri S.C., Thai P., Berkelhamer S.K., Perez M., Underwood M.A., Steinhorn R.H. (2016). Postnatal growth restriction augments oxygen-induced pulmonary hypertension in a neonatal rat model of bronchopulmonary dysplasia. Pediatr. Res..

[116] Wedgwood S., Warford C., Agvatisiri S.R., Thai P.N., Chiamvimonvat N., Kalanetra K.M., Lakshminrusimha S., Steinhorn R.H., Mills D.A., Underwood M.A. (2020). The developing gut-lung axis: Postnatal growth restriction, intestinal dysbiosis, and pulmonary hypertension in a rodent model. Pediatr. Res..

[117] Thiess T., Lauer T., Woesler A., Neusius J., Stehle S., Zimmer K.P., Eckert G.P., Ehrhardt H. (2021). Correlation of Early Nutritional Supply and Development of Bronchopulmonary Dysplasia in Preterm Infants <1000 g. Front. Pediatr..

[118] Rocha G., Guimarães H., Pereira-da-Silva L. (2021). The Role of Nutrition in the Prevention and Management of Bronchopulmonary Dysplasia: A Literature Review and Clinical Approach. Int. J. Environ. Res. Public Health.

[119] Van Kaam A.H., Rimensberger P.C. (2007). Lung-protective ventilation strategies in neonatology: What do we know—What do we need to know?. Crit. Care Med..

[120] Dreyfuss D., Saumon G. (1993). Role of tidal volume, FRC, and end-inspiratory volume in the development of pulmonary edema following mechanical ventilation. Am. Rev. Respir. Dis..

[121] Brower R.G., Matthay M.A., Morris A., Schoenfeld D., Thompson B.T., Wheeler A. (2000). Ventilation with lower tidal volumes as compared with traditional tidal volumes for acute lung injury and the acute respiratory distress syndrome. N. Engl. J. Med..

[122] Bhuta T., Henderson-Smart D.J. (2000). Elective high frequency jet ventilation versus conventional ventilation for respiratory distress syndrome in preterm infants. Cochrane Database Syst. Rev..

[123] Bollen C.W., Uiterwaal C.S., van Vught A.J. (2003). Cumulative metaanalysis of high-frequency versus conventional ventilation in premature neonates. Am. J. Respir. Crit. Care Med..

[124] Henderson-Smart D.J., Bhuta T., Cools F., Offringa M. (2003). Elective high frequency oscillatory ventilation versus conventional ventilation for acute pulmonary dysfunction in preterm infants. Cochrane Database Syst. Rev..

[125] Gommers D., Hartog A., Schnabel R., De Jaegere A., Lachmann B. (1999). High-frequency oscillatory ventilation is not superior to conventional mechanical ventilation in surfactant-treated rabbits with lung injury. Eur Respir. J..

[126] Vazquez de Anda G.F., Hartog A., Verbrugge S.J., Gommers D., Lachmann B. (1999). The open lung concept: Pressure-controlled ventilation is as effective as high-frequency oscillatory ventilation in improving gas exchange and lung mechanics in surfactant-deficient animals. Intensive Care Med..

[127] Vazquez de Anda G.F., Gommers D., Verbrugge S.J., De Jaegere A., Lachmann B. (2000). Mechanical ventilation with high positive end-expiratory pressure and small driving pressure amplitude is as effective as high-frequency oscillatory ventilation to preserve the function of exogenous surfactant in lung-lavaged rats. Crit. Care Med..

[128] Alex C.G., Aronson R.M., Onal E., Lopata M. (1987). Effects of continuous positive airway pressure on upper airway and respiratory muscle activity. J. Appl. Physiol. 1985.

[129] Cogswell J.J., Hatch D.J., Kerr A.A., Taylor B. (1975). Effects of continuous positive airway pressure on lung mechanics of babies after operation for congenital heart disease. Arch. Dis. Child..

[130] Cotton R.B., Lindstrom D.P., Kanarek K.S., Sundell H., Stahlman M.T. (1980). Effect of positive-end-expiratory-pressure on right ventricular output in lambs with hyaline membrane disease. Acta Paediatr. Scand..

[131] Faridy E.E. (1976). Effect of distension on release of surfactant in excised dogs’ lungs. Respir. Physiol..

[132] Morley C.J., Davis P.G., Doyle L.W., Brion L.P., Hascoet J.M., Carlin J.B. (2008). Nasal CPAP or intubation at birth for very preterm infants. N. Engl. J. Med..

[133] Finer N.N., Carlo W.A., Walsh M.C., Rich W., Gantz M.G., Laptook A.R., Yoder B.A., Faix R.G., Das A., Poole W.K. (2010). Early CPAP versus surfactant in extremely preterm infants. N. Engl. J. Med..

[134] Dunn M.S., Kaempf J., de Klerk A., de Klerk R., Reilly M., Howard D., Ferrelli K., O’Conor J., Soll R.F. (2011). Randomized trial comparing 3 approaches to the initial respiratory management of preterm neonates. Pediatrics.

[135] Stevens T.P., Harrington E.W., Blennow M., Soll R.F. (2007). Early surfactant administration with brief ventilation vs. selective surfactant and continued mechanical ventilation for preterm infants with or at risk for respiratory distress syndrome. Cochrane Database Syst. Rev..

[136] Schmolzer G.M., Kumar M., Pichler G., Aziz K., O’Reilly M., Cheung P.Y. (2013). Non-invasive versus invasive respiratory support in preterm infants at birth: Systematic review and meta-analysis. BMJ.

[137] (2014). Respiratory support in preterm infants at birth. Pediatrics.

[138] Kugelman A., Feferkorn I., Riskin A., Chistyakov I., Kaufman B., Bader D. (2007). Nasal intermittent mandatory ventilation versus nasal continuous positive airway pressure for respiratory distress syndrome: A randomized, controlled, prospective study. J. Pediatr..

[139] Bhandari V., Finer N.N., Ehrenkranz R.A., Saha S., Das A., Walsh M.C., Engle W.A., VanMeurs K.P. (2009). Synchronized nasal intermittent positive-pressure ventilation and neonatal outcomes. Pediatrics.

[140] Bhandari V., Gavino R.G., Nedrelow J.H., Pallela P., Salvador A., Ehrenkranz R.A., Brodsky N.L. (2007). A randomized controlled trial of synchronized nasal intermittent positive pressure ventilation in RDS. J. Perinatol..

[141] Meneses J., Bhandari V., Alves J.G. (2012). Nasal intermittent positive-pressure ventilation vs nasal continuous positive airway pressure for preterm infants with respiratory distress syndrome: A systematic review and meta-analysis. Arch. Pediatr. Adolesc. Med..

[142] Kirpalani H., Millar D., Lemyre B., Yoder B.A., Chiu A., Roberts R.S. (2013). A trial comparing noninvasive ventilation strategies in preterm infants. N. Engl. J. Med..

[143] Rüegger C.M., Owen L.S., Davis P.G. (2021). Nasal Intermittent Positive Pressure Ventilation for Neonatal Respiratory Distress Syndrome. Clin. Perinatol..

